# A Smart Home System for Information Sharing, Health Assessments, and Medication Self-Management for Older People: Protocol for a Mixed-Methods Study

**DOI:** 10.2196/12447

**Published:** 2019-04-30

**Authors:** Margaretha Norell Pejner, Wagner Ourique de Morais, Jens Lundström, Hélène Laurell, Ingela Skärsäter

**Affiliations:** 1 Department of Health and Care School of Health and Welfare Halmstad University Halmstad Sweden; 2 Technical Science School of Information Technology Halmstad University Halmstad Sweden; 3 Innovation Science School of Business, Engineering and Science Halmstad University Halmstad Sweden

**Keywords:** assessments, medication, mixed methods, older people, self-management, smart homes

## Abstract

**Background:**

Older adults often want to stay in a familiar place, such as their home, as they get older. This so-called aging in place, which may involve support from relatives or care professionals, can promote older people’s independence and well-being. The combination of aging and disease, however, can lead to complex medication regimes and difficulties for care providers in correctly assessing the older person's health. In addition, the organization of health care is fragmented, which makes it difficult for health professionals to encourage older people to participate in their own care. It is also a challenge to perform adequate health assessments and to engage in appropriate communication between health care professionals.

**Objective:**

The purpose of this paper is to describe the design for an integrated home-based system that can acquire and compile health-related evidence for guidance and information-sharing among care providers and care receivers in order to support and promote medication self-management among older people.

**Methods:**

The authors used a participatory design approach for this mixed-methods project, which was divided into four phases. Phase I, Conceptualization, consists of the conceptualization of a system to support medication self-management, objective health assessments, and communication between health care professionals. Phase II, Development of a System, consists of building and bringing together the conceptualized systems from Phase I. Phase III, Pilot Study, and Phase IV, Full-Scale Intervention, are described briefly.

**Results:**

Participants in Phase I were people who were involved in some way in the care of older adults and included older adults themselves, relatives of older adults, care professionals, and industrial partners. With input from Phase I participants, we identified two relevant concepts for promoting medication self-management, both of which related to systems that participants believed could provide guidance for the older adults themselves, relatives of older adults, and care professionals. The systems will also encourage information-sharing between care providers and care receivers. The first is the concept of the Intelligent Age-Friendly Home (IAFH), defined as an integrated residential system that evolves to sense, reason, and act in response to individuals’ needs, preferences, and behaviors as these change over time. The second concept is the Medication safety, Objective assessments of health-related behaviors, and Personalized medication reminders (MedOP) system, a system that would be supported by the IAFH, and which consists of three related components: one that assesses health behaviors, another that communicates health data, and a third that promotes medication self-management.

**Conclusions:**

The participants in this project were older adults, relatives of older adults, care professionals, and our industrial partners. With input from the participants, we identified two main concepts that could comprise a system for health assessment, communication, and medication self-management: the IAFH and the MedOP system. These concepts will be tested in this study to determine whether they can facilitate and promote medication self-management among older people.

**International Registered Report Identifier (IRRID):**

DERR1-10.2196/12447

## Introduction

### Background

Making our world more age-friendly is a key strategy to facilitate the involvement of older persons in their own care [1]. An age-friendly world supports people of all ages in actively participating in community activities and treats everyone with respect, regardless of their age. It can also enable people to stay healthy and active as they age and provide appropriate support to those who can no longer look after themselves. Remaining at home rather than moving to assisted living seems to be important to older people, even those who are ill and/or need care and supervision [2,3]. For older adults, remaining in their homes promotes independence and well-being [4].

Over half of people aged 80 years and older suffer from two or more diseases, such as diabetes, cancer, heart disease, or mental illness [5]. The presence of multiple diseases leads to an increased use of medications and the associated risk of side effects. Moreover, one out of 10 hospitalized patients experiences some kind of harm, with medication-related errors being the most common [1].

Hence, the interaction between the aging process, diseases, medication, and related side effects leads to complex health conditions that are difficult to assess and communicate [6,7]. Moreover, since care professionals are not on-site around the clock, they receive only partial information throughout the day about older adults living at home [8]. This makes assessing and making correct decisions about the older person’s health difficult. In addition, older adults themselves express their difficulty in communicating their needs and state that this is due to emotional vulnerability [9]. A further difficulty lies in the exchange of information between formal and informal caregivers. There appear to be gaps in the communication between different care professionals (eg, physicians and registered and assistant nurses) and next of kin [6,10]. This gap in communication often occurs because the various health care professionals work for different organizations, such as specialist care or outpatient care for the county council or the municipality, and lack a natural place to exchange information [6,7,10].

Research has shown that when older people self-manage their medications, there is a corresponding improvement in health status, increase in safety, and decrease in utilization and costs [8-11]. Research has also shown that medication adherence increases [8-13]. Different assistive technologies for medication compliance, such as medication dispensers, are commonly used to support independence in medication management [14]. Studies demonstrate, however, that assistive technologies for medication compliance are not suitable for older people with recurrent medication adjustments or cognitive deficiencies; this is the case because most of these devices do not include reminders or facilitate dosage adjustments and require training to operate, thus excluding the older person from the management process [8,15].

### Objectives and Research Questions

The overall aim of this project is to deliver an integrated home-based system to support and promote medication self-management among older people. More specifically, the project’s objectives are to design, develop, and evaluate an age-friendly smart home that uses smart technologies, such as sensors and medication dispensers, to collect and compile health-related evidence in order to support decision making and communication regarding medication treatment, which in turn could enhance medication self-management. The project itself includes four phases: (1) Phase I: Conceptualization, (2) Phase II: System Development, (3) Phase III: Pilot Study, and (4) Phase IV: Full-Scale Intervention. This paper focuses on Phase I and its results; Phases II, III, and IV are planned and will be described in upcoming publications.

The main research questions in the project are as follows:

1. How smart does a smart home need to be in order to increase knowledge about the older resident’s health status?
2. Which features should a smart medication-dispensing device include to support medication management among older people?
3. How could a digitalized home documentation system support health care decision making and communicate with health care professionals and older adults?
4. Can medication self-management and medication safety for older people be supported solely by single, stand-alone systems, or are integrated systems required in order to provide the expected benefits? How should such a stand-alone and/or integrated system then be implemented in practice?

## Methods

### Participatory Design

Involving older adults and caregivers in the development and evaluation of health care technologies has become increasingly relevant during recent years because their perspectives and insights can reveal needs not captured by researchers and lead to solutions that are more likely to be accepted and adopted [16]. Participatory design is a method that enables involvement, active participation, and collaboration of different stakeholders (eg, older people, relatives, caregivers, and researchers) in the codesign and coresearch activities throughout the development life cycle; the objective is a better understanding of the problem itself, reducing risks, and delivering a solution that reflects actual needs, preferences, and usage. Participatory design is also a rigorous research method and design approach [16] that draws on principles from participatory action research [17]. The method draws on the users’ *tacit knowledge* —in other words, their implicit or unarticulated knowledge as learned and transmitted through experiences and apprenticeship. Participatory design uses a variety of generative tools to establish participation [18]; the process includes the following three stages [16]:

1. Stage 1: Initial exploration of work. Observations and interviews are conducted to explore uses of the technology, routines, and aspects of the work in order to assess needs.
2. Stage 2: Discovery process. Workshops and codesign activities are organized so researchers and users can define goals and the desired outcomes of the project.
3. Stage 3: Prototyping. Researchers and users engage in the cocreation of a prototype for the solution designed in Stage 2. The resulting artifact enables further discussions and understanding of the proposed solution.

In the protocol presented in this paper, the participants’ involvement will be used throughout the research project and mixed methods [19] will be used for collecting their views; as well, there will be a particular focus on iterations in the design process.

### Research Design

#### Overview

**Figure 1:**
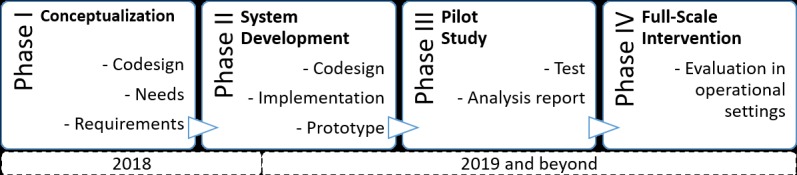
The four phases in the system development life cycle.

The research project mentioned in this work is being carried out in four phases: Phase I, Conceptualization; Phase II, System Development; Phase III, Pilot Study; and Phase IV, Full-Scale Intervention. In this research protocol, we describe Phase I more thoroughly and Phases II-IV only tentatively (see Figure 1).

#### Phase I

##### Conceptualization

This initial phase aims at enabling participants to familiarize themselves with how they can contribute and collaborate in the development of a home-based system to support and promote medication self-management among older people. This phase also includes exploring how technology can be used, work procedures, routines, safety, and any other aspects that might affect the users. In this phase, goals and values will be clarified in order to conceptualize the desired outcome of the project.

##### Participants

The participants were caregivers, the older people themselves, relatives, and the industrial partners. Caregivers from the municipal health care organizations in the municipalities of Halmstad and Hylte, Sweden, and the older people themselves were contacted through two different networks. The first network included the county council of the region of Halland, Sweden; the municipalities of Halmstad and Hylte, Sweden; and Halmstad University, Sweden, all of which share a mutual goal of collaborating in the development and introduction of eHealth in the region. A total of 13 people from this network participated: 9 females (69%) and 4 males (31%). Out of the 13 participants, 4 (31%) were registered nurses, 4 (31%) were assistant nurses, and 5 (38%) were managers. The second network included representatives from the National Organization of Pensioners from the county council of the region of Halland, Sweden (1 male representative), and from the Family Caregiver Association from Halmstad University (1 female representative). In addition, 2 industrial partners joined the project, contributing 7 participants to the study (3 participants from the first industrial partner and 4 from the second industrial partner). The first industrial partner produces medication-dispensing devices and had recently finished a pilot study in which its device was used by older persons in their homes and by care staff. The other industrial partner has developed Web-based questionnaires for collecting and communicating health care information.

##### Procedures

To explore the health care providers’ and the industrial partners’ tacit knowledge and way of working, they were initially visited at their workplaces. Thereafter, seminars were organized in order to create a shared understanding of pertinent issues and complexities related to medication management. Questions discussed were as follows: Can a technical solution be an alternative to the medication administration routines used today? What challenges do care professionals consider to be associated with medication administration? What challenges and solutions can technology bring? What ethical aspects are implied in introducing technological solutions for medication administration?

The participants were allowed to freely discuss these issues, and notes were taken. These were performed asynchronously, and 15 seminars were conducted in total. In addition to seminars, nine workshops were organized. Workshops differed from the seminars in that specific questions were asked relating to the participants’ expert knowledge and their suggested solutions.

In order to obtain additional knowledge in the field, the research group participated in two different conferences about smart homes for older people.

##### Evaluation of Phase I

It became evident to both our participants and us during Phase I that the administration of medications was complex and that current routines are sometimes contradictory. Registered nurses reported that existing solutions using different kinds of drug dispensers were not safe because all tablets cannot be divided in them, all tablets do not fit into them, and one person can have many different administration systems. This made it difficult for them to have control over the medication (eg, if any interactions and side effects occur). Registered nurses reported that existing solutions using different kinds of drug dispensers were not safe for a variety of reasons; for example, the variables offered by the dispensers in terms of administration times were often insufficient to meet the needs of the patient’s various complex medication schedules, or were simply not large enough to hold all of the patient’s medications, resulting in difficulties for nurses in tracking the sources of interactions and side effects. At the same time, for practical reasons they were forced to delegate the medication administration to an assistant nurse, who is a nonauthorized staff member. Furthermore, they pointed out that most deviations are related to incorrect medication lists and unclear prescriptions, and there is an insufficient number of people to whom they can delegate.

The assistant nurses, on the other hand, expressed a fear of making mistakes. At the same time, it appears that relatives take a great deal of the responsibility relating to medication administration. The participants realized that a technical solution could be an alternative but also expressed concerns about whether it could solve the problem. In addition, anxiety was raised about the risk of missed social contacts for the elderly person, as staff would not have to come to their homes to administer drugs to them. During the workshop and seminars, it emerged that the care staff perceived the introduction of home-based technology as something that would support them in their daily work but would also be challenging. They lacked confidence in using technology; in addition, they feared that the older person would have fewer visits and, thus, less human contact.

With input from the participants, we identified two main concepts that could comprise a system to collect and compile health-related evidence to support decision making and communication regarding medication treatment, which in turn could enhance medication self-management: the *Intelligent Age-Friendly Home* (IAFH) and the *Medication safety, Objective assessments of health-related behaviors, and Personalized medication reminders* (MedOP) system.

#### Intelligent Age-Friendly Homes

Smart homes integrate home-based, network-enabled technologies that cannot only automate and control devices in the home, but also monitor the household, learn the habits and preferences of the residents over time to anticipate their needs, and take actions automatically or with minimal guidance from residents [20-22]. The extent of the autonomy of a smart home is what determines its “smarts” [22]. Over the past decades, there has been an increased interest in exploring, developing, and using smart home technologies in health care. For example, the pervasive technological infrastructure provided by smart homes has been explored in a number of projects supporting Ambient Assisted Living, which aims to provide intelligent and transparent forms of monitoring and assistance for older and disabled individuals [23].

The World Health Organization also recognizes the impact of the environment, such as home and community, on people's health and well-being at different stages of life, and particularly in later years. It proposed creating an age-friendly environment as one of its five priority areas for action concerning ageing and health, saying that such an environment “combats ageism, enables autonomy, and supports Healthy Ageing,” which is defined as the “process of developing and maintaining the functional ability that enables well-being in older age” [5].

In this project, we propose the concepts of the IAFH and define them as integrated residential systems that evolve to sense, reason, and act according to individual needs, preferences, and behaviors as these change over time; in other words, a smart home that is attractive to people when they are young and is supportive of them as they age [24]. We also propose the development and evaluation of an IAFH to support medication self-management for older people. More specifically, the IAFH is envisioned to support functional monitoring (eg, activities and sleep), cognitive and sensory assistance (eg, medication reminders and drug dispensers), and personal interaction (eg, communication). Although these are the main categories of health-related smart home technologies [25], the novelty in the proposal lies in the integration of those functionalities to support medication self-management.

#### Medication Safety, Objective Assessments of Health-Related Behaviors, and Personalized Medication Reminders System

The MedOP system encompasses the integration of three different stand-alone systems with the objective of supporting medication safety, objective health assessment, and personalized medication reminders within an IAFH. The MedOP system integrates the following three subsystems.

##### Home Sensors

Various kinds of sensors, including passive infrared sensors, switch sensors, pressure sensors, smoke and gas sensors, cameras, and more, can measure different physical, motion, contact, and presence properties within a smart home. At Halmstad University, the Halmstad Intelligent Home is a fully functional, campus-based, two-room apartment that is densely outfitted with sensors and actuators. The Halmstad Intelligent Home uses a research-based, database-centric system architecture that serves as a platform for the development of smart homes with applications in health care. The system architecture focuses on different quality attributes, such as interoperability, scalability, dependability, security, and privacy.

##### Digitalized Home Documentation

Handling and communicating health-related information can be difficult. To overcome this issue with the use of Digitalized Home Documentation, which standardizes health-related information from the Home Sensors, we envision more accurate health assessments and communication between care receivers and caregivers, thus increasing the patient’s participation in his or her own health care. We plan to achieve this standardization with the integration of nursing taxonomies. These terminologies and tools are expected to support nurses in making diagnoses and to improve education, communication, and reporting among caregivers at work. We plan to develop this component in collaboration with one of our industrial partners.

##### Digitalized Medication Dispenser

Physical and cognitive impairments can prevent individuals from using assistive devices or remembering the medication regimen. This part of the proposal will be developed in collaboration with our second industrial partner; this partner has previously developed a pill dispenser that can manage and schedule complex medication regimes remotely, using a cloud-based management system via different communications technologies to ensure connectivity. The proposed Digitalized Medication Dispenser system will be enhanced with objective information collected by the Home Sensors and the Digital Home Documentation systems, so that personalized scheduling and context-based medication reminders can be provided.

**Figure 2:**
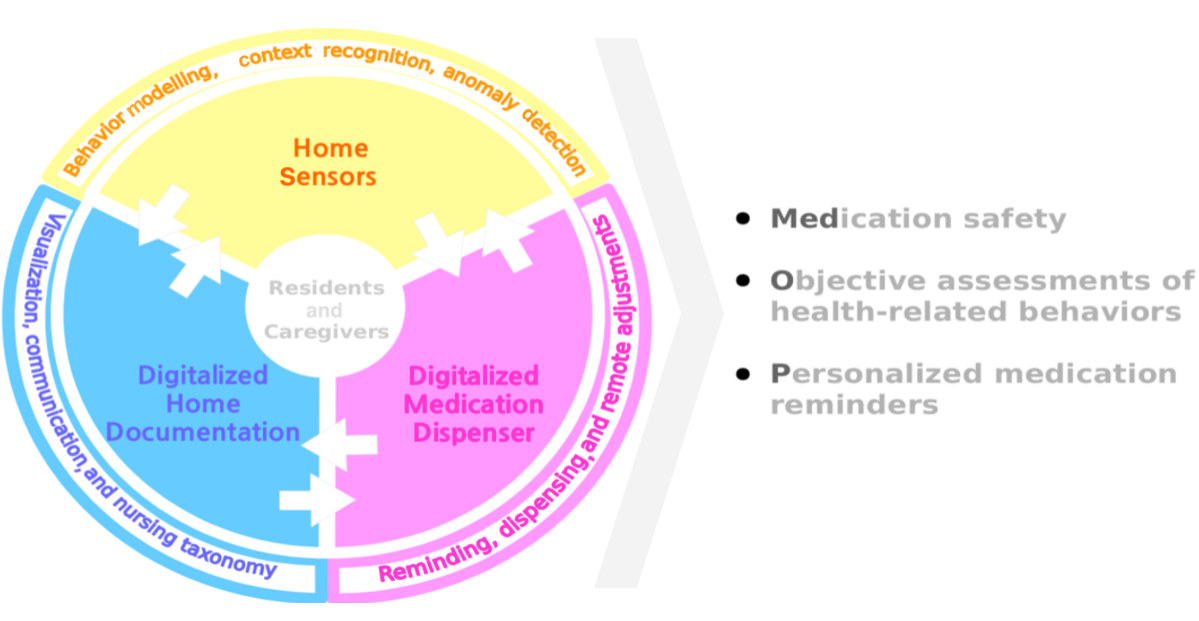
The Medication safety, Objective assessments of health-related behaviors, and Personalized medication reminders (MedOP) system with its three integrated systems.

The three developed and integrated systems—Home Sensors, Digitalized Home Documentation, and Digitalized Medication Dispensers—form the MedOP system (see Figure 2), which will provide objective information to the care staff, allowing them to improve their decision making. The information provides an objective summary of a person’s in-home activity: daily routines; current and expected location; medication dispensing; amount and quality of sleep; weight; bathroom usage; and level of, and change in, activities. The MedOP system is to be developed in a way that includes older adults in the care process by raising their awareness of their health and behaviors. Moreover, the MedOP system will help older people remember and administer their medication by tailoring medication reminders to their actual location and behaviors, as well as to their regimen.

#### Phase II

##### Development

The conceptualization of a smart system for collecting and compiling health-related evidence, supporting decision making and communication, and improving medication self-management developed in Phase I will be used to build a prototype in Phase II. This will take place in a laboratory environment at Halmstad University and the users will be involved.

##### Participants

Participants will be the same as in Phase I (ie, caregivers from the municipal health care organization, the older people themselves from two different ongoing networks, and the industrial partners); there will be 22 persons in total.

##### Procedures

Along with our participants, we will identify technical and nontechnical risks and ethical standpoints, as well as quality, design, and commercial issues. In seminars, risks and ethical standpoints will be discussed based on the following questions: Do you feel confident in your ability to use a technical solution for medication administration? How will your work situation be affected? How do you feel about a technical solution that would complement staff? These issues and those that arise during the course of the project must be considered. Other techniques will also be used to design the tool. First, a paper prototype will be used to design and test the user interface. A beta version of the paper prototype will then be built and tested in a laboratory environment. In addition, an application programming interface will be defined to facilitate and secure communication between systems. We will also identify and apply a method for software integration. Finally, the technology will be tested individually and as an integrated system in a home-like laboratory environment at Halmstad University.

#### Phase III

##### Pilot Study

In Phase III, we will pilot the implementation of the MedOP system. In the pilot study we will evaluate the functionality of the technology, adjustments, and feasibility. This will be performed by installing the MedOP system at the home of the elderly to test it in a real environment.

##### Participants

A total of 10 persons, 65 years or older, who are utilizing five or more different medications and have home help service and home nursing care will be selected. The older person will be assessed by their registered nurse to determine if they have the ability to manage their medication by themselves. The older persons will be recruited via the National Organization of Pensioners and the Family Caregiver Association. The registered and assistant nurses who are involved in the care and service of the 10 older persons will also be included in the study; this will bring the number of participants up to 15-20 persons. The registered and assistant nurses will be recruited via their managers.

##### Procedures

Quantitative data will be collected from the 10 older persons at baseline and at one follow-up session after 3 months using the MedOP system. The following instruments will be used for each measure:

1. Medication adherence: Morisky Medication Adherence Scale [26].
2. Well-being: Personal Well-being Index-Adult [27].
3. Self-management: *30*-item Self-Management Ability Scale [28].
4. Life satisfaction: Satisfaction With Life Scale [29].
5. Serenity: Serenity Scale [30].

Qualitative data will be collected at the end of Phase III. Individual interviews will be performed with the 10 older adults who are using the MedOP system, and focus groups will be used with the nursing staff. Each focus group will consist of 6-8 participants, with separate groups for registered nurses and assistant nurses. The interviews will be analyzed using content analysis [31] and grounded theory [32,33]

#### Phase IV

##### Full-Scale Intervention

Phase IV aims to test and evaluate the MedOP system in a full-scale intervention.

##### Participants

All persons 65 years or older in the two municipalities participating in the study who are utilizing five or more different medications and have home help service and home nursing care will be selected and offered the opportunity to participate in the study. The older persons will be recruited via the National Organization of Pensioners and the Family Caregiver Association. The older person will be assessed by their registered nurse to determine if they have the ability to manage their medication by themselves. The registered and assistant nurses who are involved in the care and service of the older persons will also be included in the study.

##### Procedures

In the full-scale study, the same procedures as in Phase III will be used. By evaluating the MedOP system intervention, using both quantitative and qualitative data, the follow-up of both perceived and practical challenges will be enabled.

### Ethics

Participants in Phase I were recruited via networks whose purpose is to improve and develop working methods and working conditions for care providers but also quality of care for care receivers in the municipalities. Involvement in this project can thus be seen as part of their occupational development.

Phase I of the project is not considered to have exposed the participants to danger or discomfort. Participation has been on a voluntary basis; oral and written information has been given before each session regarding the purpose of the study and that the data may be used in research.

In Phases II-IV, ethical issues are involved in the different activities of their procedures. Since the implementation of technology and high-tech care will occur in home settings, researching the unintended and ethical effects resulting from the implementation is important.

During Phase II, the researchers will collect qualitative observational and interview data in a simulated laboratory setting on healthy volunteer subjects to identify both ethical and unintended effects resulting from the implementation of new health and welfare technologies.

In Phases III and IV, a pilot study and full-scale study using similar data collection methods as in Phase II will be focused on subjects who are actually using new health and welfare technologies in their homes. As these will be elderly people needing additional support in the home, they will be treated by the researchers as vulnerable adults. In these latter phases, in addition to data collected about unintended and ethical consequences, other sensitive data will be gathered, including data on well-being, life satisfaction, and medication regimes. Special consideration will be made to limit risks associated with working with vulnerable subjects, especially in the areas of privacy and informed consent.

Approval from the Regional Committee for Medical and Health Research Ethics shall be completed to ensure that ethical aspects are handled correctly, according to Swedish law.

## Results

For this study, we identified and included 22 participants that represent four different stakeholder groups (ie, caregivers from the municipal health care organizations, the two groups of older people from two different ongoing networks, and the industrial partners). The project was funded in 2016-2017 and enrollment for Phase I was completed in 2018. Parts of the MedOP system are in use and the continued work in Phase II will further develop and merge the different systems—Home Sensors, Digitalized Home Documentation, and Digitalized Medication Dispensers—with each other. Ethical approval is currently underway, and the first results are expected to be submitted for publication in mid-2020.

## Discussion

The use of participatory design [16] gave us the opportunity to explore different users’ perspectives and needs and propose a person-centered solution to a common and complex problem among older people, which is self-managing their medication. To better understand the problem, current approaches, and actual needs, we involved different stakeholders at the very early stages of this project. Moreover, it was expected that such an approach would lead to a consensus toward how technology could be employed to overcome the problem. However, although the care staff participating in the seminars and workshop identified and recognized the benefits of home-based technologies in their work, they also expressed concerns regarding the introduction of new methods and routines into their work. The participating health care staff also expressed a lack of confidence in using technology and feared that the older persons would have fewer visits, resulting in less human contact. Research has shown that this kind of attitude can be a barrier to the introduction of new technology systems [26]. Haken, Allouch, and van Harten [34] conclude that when introducing advanced medical technologies into the home, it is important to also provide education, clear guidelines, and information about risk management and patient safety at the same time. Developing and providing documentation and training regarding the system are requirements for Phases II, III, and IV.

In addition, and in order to ensure that the end-project results reach the intended market and users, the close collaboration with our industrial partners provided critical insights and perspectives regarding technology development and commercialization in the health care domain. We therefore argue that it is important to both understand the logic and incentives from the supply side (ie, the firms that will commercialize the technologies) and the demand side (eg, the health care professionals and older persons who will be the end users of the technologies and solutions) when operating in a networked health care system [35,36]. Moreover, it is also critical to understand who will pay for the health care innovation, both within the country and internationally, in order to ensure that the innovation will actually be implemented. We therefore propose that we also add a business model [37] perspective for our future research agenda to meet the supply and demand of innovations in health care, with a particular focus on value capture from innovations [38-40].

## Abbreviations

IAFH: Intelligent Age-Friendly Home
MedOP: Medication safety, Objective assessments of health-related behaviors, and Personalized medication reminders
